# Survivorship care plans and adherence to breast and cervical cancer screening guidelines among cancer survivors in a national sample

**DOI:** 10.1007/s00520-024-08986-2

**Published:** 2024-11-18

**Authors:** Marco Santos-Teles, Ganesh Modugu, Isabel C. Silva, Elisa V. Bandera, Mridula George, Bo Qin, Jonathan Smith, Ruth Stephenson, Malcolm D. Mattes, Mariam F. Eskander

**Affiliations:** 1 Rutgers New Jersey Medical School, Newark, NJ USA; 2 Cancer Epidemiology and Health Outcomes, Rutgers Cancer Institute of New Jersey, New Brunswick, NJ USA; 3 Division of Medical Oncology, Rutgers Cancer Institute of New Jersey, New Brunswick, NJ USA; 4 Division of Surgical Oncology, Rutgers Cancer Institute of New Jersey, New Brunswick, NJ USA; 5 Division of Radiation Oncology, Rutgers Cancer Institute of New Jersey, Newark, NJ USA; 6 Rutgers Cancer Institute of New Jersey, Robert Wood Johnson Medical School, 195 Little Albany St., New Brunswick, NJ 08903 USA

**Keywords:** Survivorship care, Cancer screening, Breast cancer, Cervical cancer

## Abstract

**Purpose:**

The impact of the components of survivorship care plans on adherence to cancer screening guidelines among cancer survivors is limited. We examined the association of receipt of treatment summaries, follow-up instructions, and type of doctor providing survivorship care with adherence to breast cancer screening (BCS) and cervical cancer screening (CCS) guidelines in female cancer survivors.

**Methods:**

A cross-sectional analysis using Behavioral Risk Factor Surveillance System (BRFSS) data from 2014, 2016 and 2018 was conducted. BCS and CCS-eligible women were aged 40–74 and 30–64, respectively. BCS adherence was defined as a mammogram within 2 years and CCS adherence as a pap smear within 3 years or HPV test within 5 years. Univariate analysis with chi-square and multivariable logistic regression are reported.

**Results:**

5,001 BCS and 3,014 CCS-eligible survivors were identified. In the BCS group, recipients of treatment summaries and follow-up instructions were significantly more adherent with BCS (84.1% vs. 77.4%; 83.4% vs. 74%, respectively, *p* < 0.001). In the CCS group, recipients of follow-up instructions were significantly more adherent with CCS (78.1% vs. 67.7%, *p* < 0.001). In both groups, there was no significant difference in BCS or CCS based on type of physician providing care (*p* = 0.087). On multivariate analysis, receipt of follow-up instructions was the only factor significantly associated with BCS (OR:2.81; 95%CI:1.76–4.49) and CCS (OR:3.14; 95%CI:1.88–5.23).

**Conclusions:**

Follow-up instructions, as part of survivorship care plans, have the strongest association with BCS and CCS among female cancer survivors. Additional research should focus on improving the distribution of survivorship care plans, particularly follow-up instructions, as a method to increase BCS and CCS among cancer survivors.

## Introduction

Cancer survivors are a rapidly growing population in the United States, as malignancies are diagnosed at earlier stages and treatment advances increase the probability of cure [1]. In January 2022, there were about 18.1 million cancer survivors in the U.S., encompassing 5.4% of the overall population; this number is expected to grow to 22.5 million by 2032 and to 26 million by 2040 [1]. This trend poses a critical question to healthcare providers: how do we provide optimal longitudinal care for this unique, growing population?

Subsequent age-appropriate screening for a new primary malignancy among cancer survivors is a quintessential component of longitudinal care [2, 3]. Compared to the general population, cancer survivors are at increased risk of developing an additional primary cancer or recurrent cancer due to persistent risk factors [2–4]. Prior studies have reported both higher [5, 6] and lower [7, 8] screening utilization of cancer survivors compared to cancer-free controls, though the most recent meta-analyses on this topic suggests that survivors are more likely to undergo screening [9, 10]. Despite this, the overall screening rates among cancer survivors are still suboptimal, especially for cervical cancer [11], and there are many high-risk subgroups (e.g. racial/ethnic minorities, low-income individuals) that are less likely to be screened [12, 13]. Given the increased subsequent cancer risk and suboptimal screening rates, it is critical to explore how to increase cancer screening uptake among cancer survivors, particularly among those who face barriers to healthcare.

“Survivorship care” is a broad concept that incorporates numerous factors related to physical health into the longitudinal care of cancer survivors. Survivorship care plans (SCP) were introduced as an effort to standardize and bridge future healthcare for survivors [14]. The National Cancer Institute (NCI) defines a SCP as a “detailed plan given to a patient after treatment ends that contains a summary of the patient’s treatment, along with recommendations for follow-up care [15].” The American Society of Clinical Oncology (ASCO) has developed a standardized SCP form that includes details regarding the patient’s specific diagnoses, the timing and type of therapies used, potential adverse effects of therapy, the recommended type and frequency of follow-up, the coordinating provider(s) for follow-up care, and suggestions for a balanced lifestyle. Despite recommendations for their use and ease of implementation, the value of SCPs has been controversial, and their overall benefits to patients remain unclear [16, 17]. While some studies have shown how SCPs as a whole can contribute to the health-related behaviors and cancer surveillance rates for cancer survivors, an understanding of which of these specific components contribute to increased screening adherence is limited, particularly for female cancer survivors [18–20]. This study examines the association between specific aspects of survivorship care plans (i.e. instructions for follow-up care and summary of treatments), and physician providing survivorship of care, and adherence to breast cancer screening (BCS) and cervical cancer screening (CCS) guidelines in female cancer survivors.

## Methods

### Data source and population

In this cross-sectional analysis, data was combined from the 2014, 2016 and 2018 Behavioral Risk Factor Surveillance System (BRFSS). BRFSS collects data via telephone survey from all 50 states in the U.S. regarding health-related behaviors, prevalence of chronic diseases, and use of preventive services [21]. The surveys are conducted annually with a core common questionnaire that is collected in all states along with optional modules administered only in select states. The core questionnaire consists of questions about socioeconomic status, demographic information, health status, chronic diseases, and cancer screening. The optional modules include questions about several topics of interest to the public health of different states, including a module on cancer survivorship. The cancer survivorship module has questions regarding care provided in the post-treatment period for patients who were diagnosed with cancer in the past. Participants with a cancer history are identified with two questions in the core chronic disease module: “Has a doctor, nurse, or other health professional ever told you that you had any of the following? For each, tell me ‘Yes’, ‘No’, or you’re ‘Not sure’: 1) (Ever told) you had skin cancer? and 2) (Ever told) you had any other types of cancer?” Participants identified with a history of cancer via these questions are later asked questions from the survivorship module in states that offer them. Seven states included the cancer survivorship module in 2014, including Alaska, Iowa, Mississippi, Missouri, Nebraska, Wisconsin, and Ohio. Eleven states opted to include the cancer survivorship module in the 2016 questionnaire, including Idaho, Indiana, Louisiana, Michigan, Missouri, South Dakota, Virgin Islands, Wisconsin, Kansas, and Nebraska. Seven states opted to include the cancer survivorship module in the 2018 questionnaire, including Delaware, Indiana, Michigan, Missouri, New Jersey, South Dakota, and Kansas. Most states ask breast and cervical cancer screening questions biennially, with the great majority of states reporting data on even years. Data from 2020 was not included in this study due to the potential impact of the Covid-19 pandemic on cancer screening [22].

### Inclusion and exclusion criteria

The patient population for this study included women aged 30–74 who completed the BRFSS questionnaire in a state with the optional cancer survivorship module. Women who self-reported a history of one or more cancers and completed treatment were included. Treatment completion was established with the following question in the survivorship module: “Are you currently receiving treatment for cancer? [By treatment, we mean surgery, radiation therapy, chemotherapy, or chemotherapy pills].” Those who answered “No, I have completed treatment” were included in the analysis. Women who had skin cancer other than melanoma were excluded from the analysis.

### Study variables

**Fig. 1:**
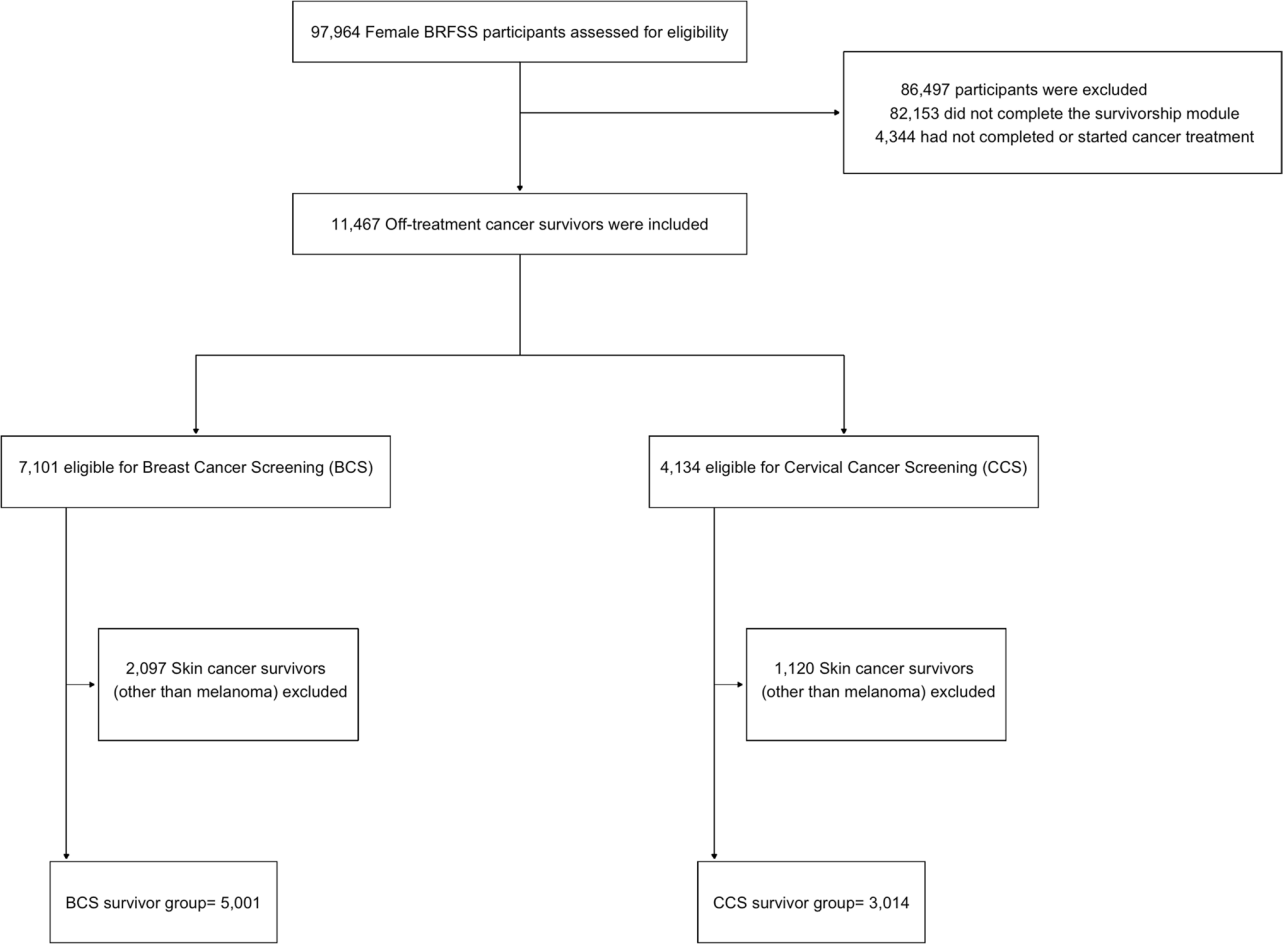
Consort diagram

We conducted separate analyses in the BCS-eligible group and a CCS-eligible (CCS) group (Fig. 1).

The criteria for cancer screening were based on the U.S Preventative Services Task Force (USPSTF) guidelines [23, 24]. In 2014, the USPSTF did not recommend routine screening for women aged 40–49 [25], though other organizations continued to support screening for this age group, including the American College of Obstetrics and Gynecology (ACOG) and American Cancer Society (ACS) guidelines [26–29]. The USPSTF later updated their recommendations to support selective BCS screening for women aged 40–49 in 2016 [24].

BCS-eligible women were aged 40–74, and CCS-eligible women were 30–64. BCS completion was defined as receipt of a mammogram within the last 2 years, and CCS completion was defined as receipt of a pap smear within the last 3 years or an HPV test within the last 5 years, or both.

The survivorship care module asks specifically about two components of survivorship care plans: instructions for follow-up care and summary of treatments. BRFSS defines follow-up instructions as instructions from a health professional about where to return and who to see for routine cancer check-ups after completing cancer treatment, and the summary of treatments as a written summary of all cancer treatments provided by a health professional [30]. Additional questions are asked to determine those who received follow-up instructions, and whether this was received in a written format [30]. Receipt of follow-up instructions included both written and unwritten instructions provided by a health professional. Receipt of a formal SCP was defined as patients who specifically received both written follow-up instructions and a written treatment summary. The BRFSS additionally asks the type of doctor providing the majority of the cancer survivors’ health care, with choices including “Family Practitioner,” “General Practitioner-Internist,” “Cancer Surgeon,” “Medical Oncologist,” “Radiation Oncologist,”” Gynecologic Oncologist,” “Urologist,” “Plastic Surgeon,” and “Other.” These choices were consolidated into three categories: primary care physician (general and family practitioner), specialist (medical, surgical, radiation, or gynecologic oncologist, urologist or plastic surgeon) and other (not further specified in BRFSS). Demographic information regarding age, race and ethnicity, education, having insurance at time of survey and at time of treatment, employment, income, partner status, BMI, and smoking were collected.

### Statistical analysis

Univariate analysis was performed using chi-square to determine differences in BCS and CCS completion across different subgroups. Multivariable analysis was performed using binary logistic regression to determine which components of survivorship care plans were associated with BCS or CCS completion. These models were adjusted for sociodemographic factors and health behaviors with a known clinical significance on cancer screening behaviors [31–34], including race and ethnicity, education, having insurance at time of survey, employment, income, partner status, BMI, and smoking.

Another multivariate model was created to determine socioeconomic characteristics associated with increased receipt of follow-up instructions. All socioeconomic characteristics included as covariates in the first model predicting screening were included (race/ethnicity, education, employment, insurance, and income) in the second model as potential predictors of receipt of follow-up instructions. Insurance at time of diagnosis (vs. at time of survey) was chosen for the second model because it better reflects coverage at time of receipt of survivorship care plan.

All analyses were conducted using complex sampling methods with BRFSS-provided sampling weights to account for non-coverage and sampling bias among the population [35]. An alpha level of 0.05 was considered statistically significant. SPSS version 28 (IBM Corp., Armonk, NY) was used for statistical analysis, and R statistical software version 4.3.1 (R Core Team, 2023) was used to produce all figures.

## Results

A total of 60,699 people responded to the 2014 questionnaire, 66,499 to the 2016 questionnaire, and 44,991 to the 2018 questionnaire. The combined database had a total of 172,189 participants, among which 97,964 were female. 5,001 BCS-eligible and 3,014 CCS-eligible survivors were identified, as shown in Fig. 1. Participant demographics are shown in Table 1. For both groups, the majority were white, highly educated, had insurance coverage, and had a partner. Most were also overweight or obese, and about half of participants were current or former smokers. Within the BCS group, 88.9% were non-Hispanic white, 42.0% were employed, and 54.7% had an income of less than $50,000. 36.2% of this group had a history of breast cancer, followed by cervical cancer (13.9%) and melanoma (13.9%). Within the CCS group, 87.7% were non-Hispanic white, 55.1% were employed, and 50.5% had an income of less than $50,000. Breast cancer was also the most common cancer reported (29.2%), followed by cervical cancer (21.5%), and melanoma (13.8%).

**Table 1:** Demographics

		BCS-Eligible Survivors *n* (%)	CCS Eligible-Survivors *n* (%)
*n*		5,001	3,014
*n* weighted		546,918	425,268
Age	30–39	-	56,443 (13.3)
	40–49	96,415 (17.7)	96,415 (22.7)
	50–59	168,034 (30.7)	168,033 (39.5)
	60–64	104,377 (19.1)	104,377 (24.5)
	65–75	178,093 (32.6)	-
Race / Ethnicity	White	482,240 (88.9)	369,913 (87.7)
	Black/AA	34,485 (6.4)	25,991 (6.2)
	Hispanic	11,555 (2.1)	11,812 (2.8)
	Other	14,284 (2.6)	14,105 (3.3)
Education	< High School	45,018 (8.2)	35,086 (8.3)
	High School/GED	176,216 (32.2)	129,148 (30.4)
	> High School	325,252 (59.5)	260,707 (61.4)
Employment	Employed	228,968 (42.0)	233,768 (55.1)
	Unemployed	18,607 (3.4)	23,090 (5.4)
	Out of labor force	297,522 (54.6)	167,383 (39.5)
Income	< $25,000	135,997 (28.4)	111,437 (29.0)
	$25,000-$50,000	126,015 (26.3)	82,719 (21.5)
	$50,001-$75,000	81,365 (17.0)	62,894 (16.4)
	> $75,000	135,363 (28.3)	127,030 (33.1)
Partnered status	Partnered	337,364 (61.9)	267,027 (62.9)
	Not partnered	207,887 (38.1)	157,394 (37.1)
Insurance Coverage	Covered	524,654 (96.0)	395,898 (93.2)
	Not Covered	21,858 (4.0)	28,985 (6.8)
BMI	Underweight (< 18.5)	12,740 (2.5)	11,705 (3.0)
	Normal Weight (18.5–24.9)	153,000 (30.2)	127,719 (32.5)
	Overweight (25–29.9)	157,895 (31.1)	115,670 (29.4)
	Obese (> 30.0)	183,377 (36.2)	138,194 (35.1)
Smoking	Current	109,717 (20.1)	109,563 (25.8)
	Former	163,685 (30.0)	106,669 (25.1)
	Never	272,018 (49.9)	208,233 (49.1)
Adherence to Screening Guidelines %		81.1%	75.2%

76.4% of BCS-eligible and 77.5% of CCS-eligible survivors received follow-up instructions, of which 80.4% and 82.0% were written, respectively. 50.1% of BCS-eligible and 50.4% of CCS-eligible survivors received a treatment summary. 38.4% of BCS-eligible survivors and 39.4% of CCS-eligible survivors received a formal SCP. 68.4% of BCS-eligible survivors received the majority of their care from a primary care physician, 18.8% by a specialist, and 12.8% by another type of physician not listed (“other”). 63.6% of CCS-eligible survivors received the majority of their care from a primary care physician, 22.0% by a specialist, and 14.4% by another type of physician not listed. 81.1% of the BCS eligible survivors were guideline adherent with their mammogram, and 75.2% of the CCS eligible survivors were guideline adherent with their pap smear and/or HPV testing.

**Fig. 2:**
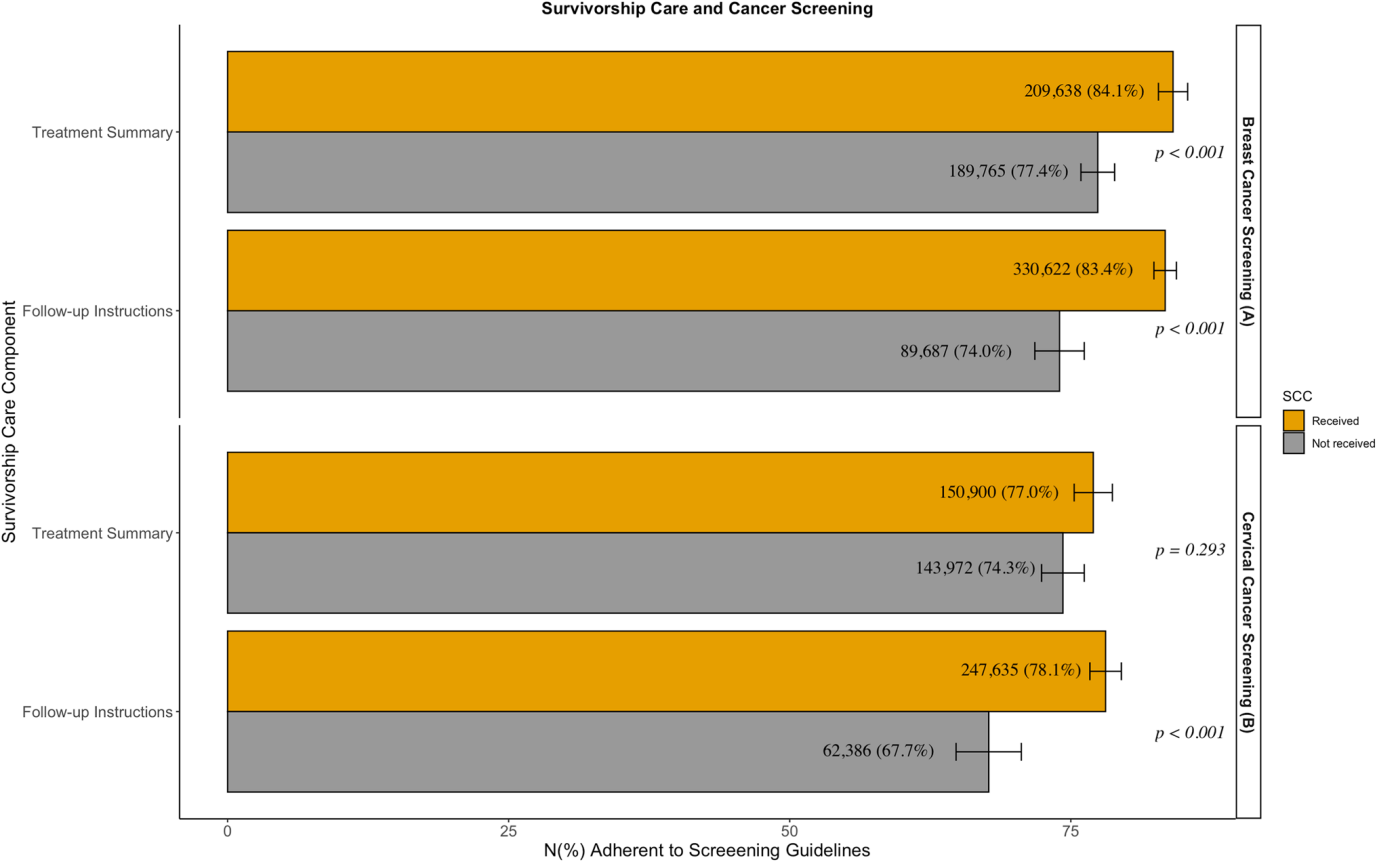
Univariate analysis of receipt of survivorship care components vs. guideline adherence to **A**) Breast cancer screening and **B**) Cervical cancer screening. *error bars represent standard error of the percentages. **reported N are weighted counts

**Fig. 3:**
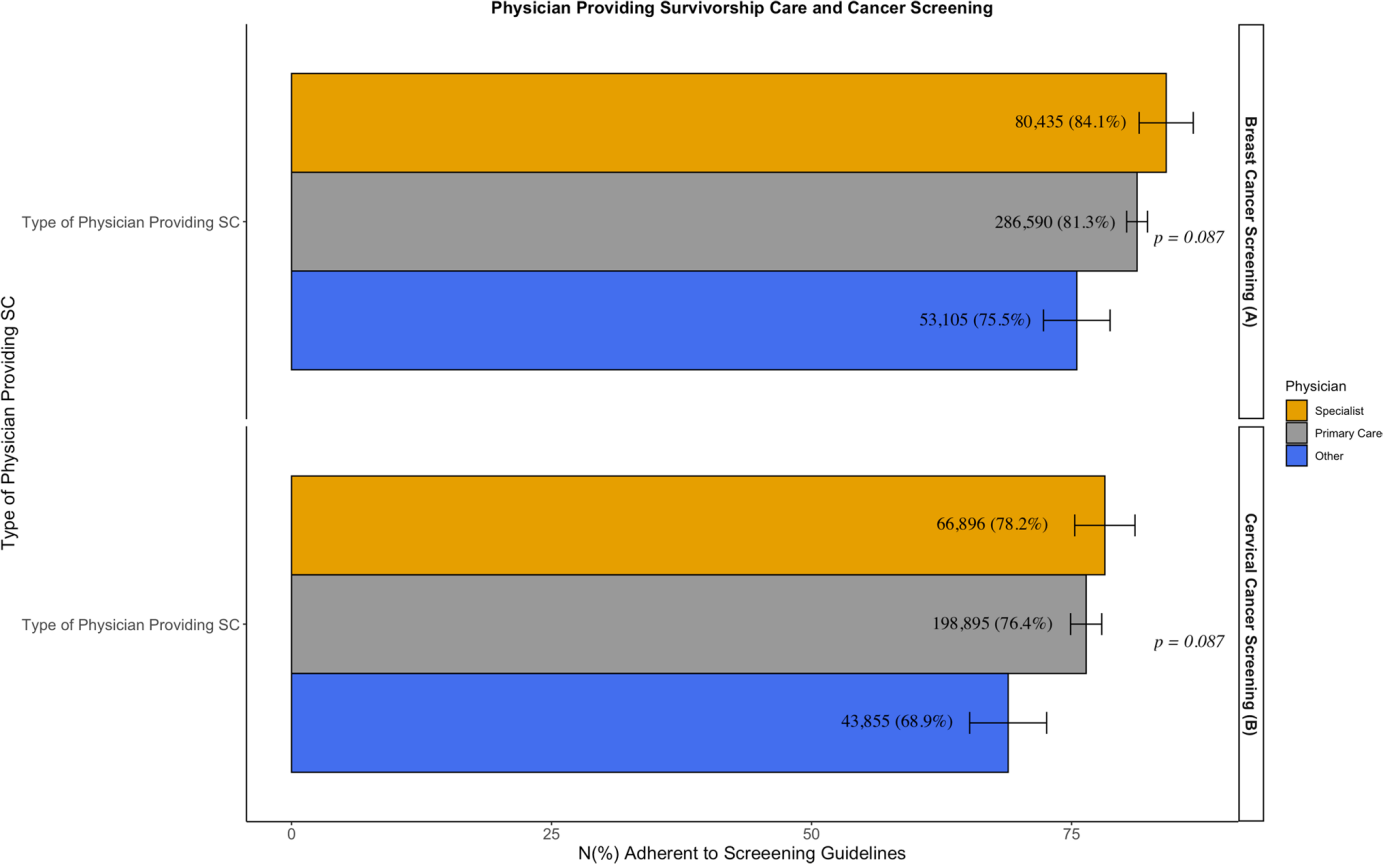
Univariate analysis of physician providing survivorship care vs. guideline adherence to **A** Breast cancer screening and **B** Cervical cancer screening. *error bars represent standard error of the percentages. **reported N are weighted counts

Among BCS-eligible survivors, recipients of a treatment summary (84.1% vs. 77.4%, *p* < 0.001) and follow-up instructions (83.4% vs. 74%, *p* < 0.001) were significantly more guideline adherent to BCS than non-recipients. There was no significant difference in guideline adherence between those receiving written vs. unwritten follow-up instructions (83.6% vs. 81.9%, *p* = 0.507). Among CCS-eligible survivors, there was no significant difference in CCS for patients who did and didn’t receive a treatment summary CCS (*p* = 0.293), but recipients of follow-up instructions were significantly more guideline adherent with CCS (78.1% vs. 67.7%, *p* < 0.001). There was also no difference in guideline adherence between those receiving written vs. unwritten follow-up instructions (77.8% vs. 78.1%, *p* = 0.942). In both groups, there was no significant difference in screening based on the type of physician providing survivorship care (*p* = 0.087). Results of the univariate analysis are shown in Figs. 2 and 3.

**Fig. 4:**
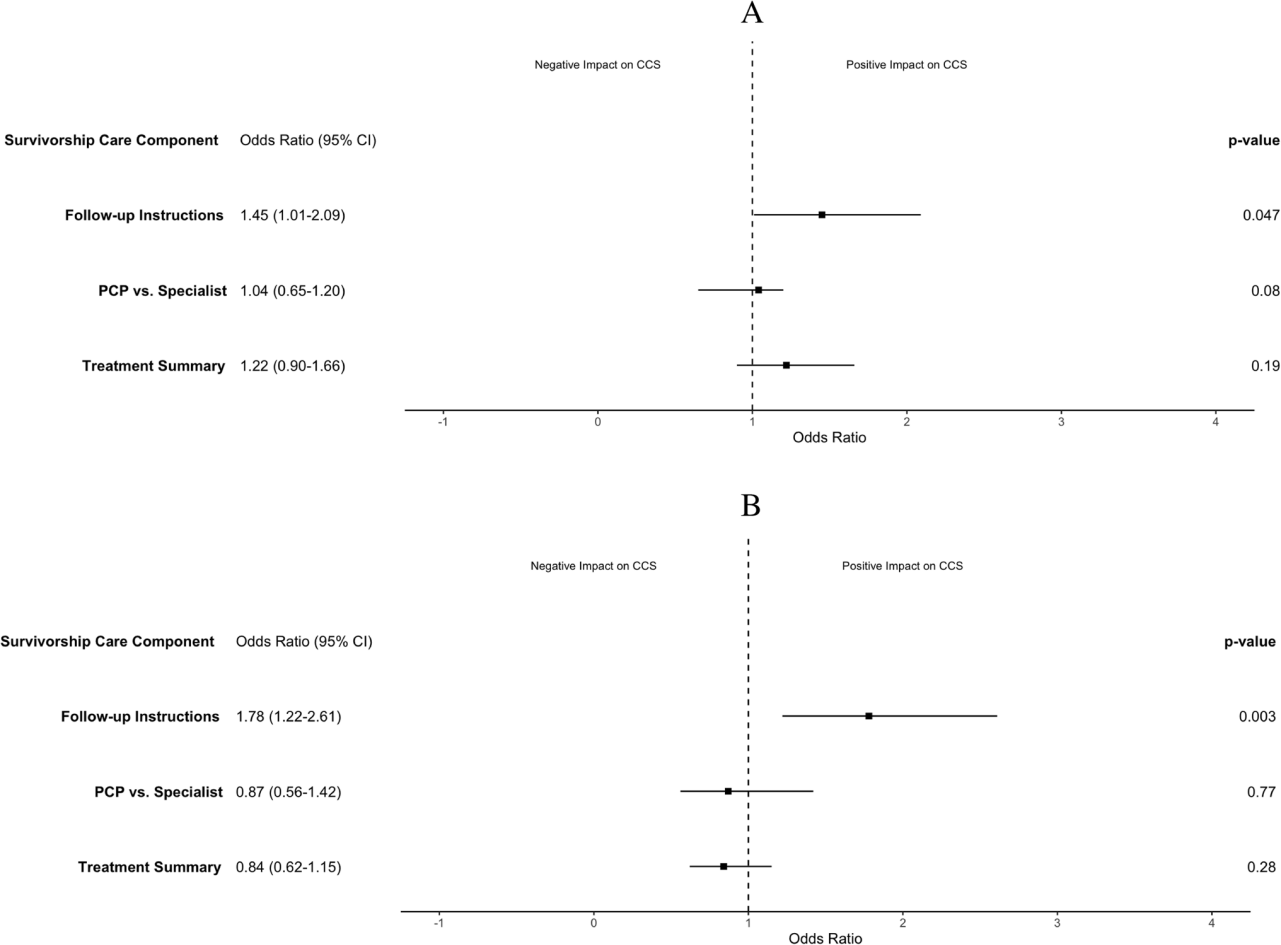
Associations of characteristics of survivorship care with guideline adherence to **A** Breast cancer screening and **B** Cervical cancer screening. **Covariates: race and ethnicity, education, having insurance at time of survey, employment, income, partner status, BMI, and smoking

On multivariable analysis, receipt of follow-up instructions was significantly associated with greater odds of both BCS (odds ratio [OR], 1.45; 95% CI, 1.01–2.09) and CCS (OR, 1.78; 95% CI, 1.22–2.61). In addition, having insurance at time of the survey was significantly associated with guideline adherence to BCS (OR, 2.91; 95% CI, 1.54–5.50). Being a non-Hispanic Black survivor compared to a non-Hispanic white survivor (OR, 5.77; 95% CI, 2.33–14.3) and having an income > $75,000 compared to < $25,000 (OR, 2.22; 95% CI, 1.47–3.36) were significantly associated with guideline adherence to CCS. Figure 4 shows the results of the multivariable model predicting adherence to guidelines for BCS and CCS.

**Fig. 5:**
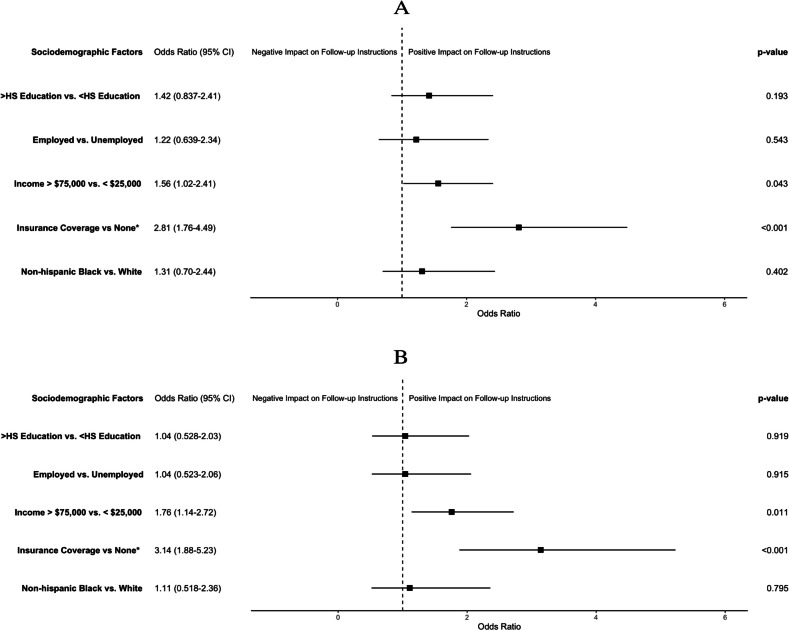
Sociodemographic factors associated with receipt of follow-up care instructions among survivors eligible to receive breast cancer (**A**) and cervical cancer (**B**) screening. *at time of cancer diagnosis. **Covariates: insurance at time of survey, partner status, BMI, and smoking

Because receipt of follow-up care was found to be the stronger predictor of adherence to screening guidelines, we further evaluated socioeconomic factors associated with receipt of follow-up care instructions. In the BCS-eligible group, those with insurance coverage at the time of diagnosis were more likely to receive follow-up care instructions (78.4% coverage vs. 57.7% no coverage, *p* < 0.001). Participants in higher income brackets (80.3% [> $75,000] vs. 70.2% [< $25,000], *p* < 0.001) and with higher education levels (78.1% [> High School] vs. 75.9% [HS] vs. 65.7% [< HS], *p* = 0.010) were also more likely to receive follow-up care instructions. There was no statistically significant association of employment status and race/ethnicity with receipt of follow-up care instructions (*p* = 0.156 and *p* = 0.797, respectively). On multivariable models predicting receipt of follow-up instructions, insurance coverage during cancer care was significantly associated with receipt of follow-up instructions for both the BCS (OR, 2.81; 95% CI, 1.76–4.49) and CCS groups (OR, 3.14; 95% CI, 1.88–5.23). An income > $75,000 compared to < $ 25,000 was also significantly associated with receiving follow-up instructions for both the BCS (OR, 1.56; 95% CI, 1.02–2.41) and CCS group (OR, 1.76; 95% CI,1.14–2.72). Race, level of education, and employment status were not significantly associated with receipt of follow-up instructions for either group. Figure 5 shows the results of the multivariate models.

## Discussion

As the number of cancer survivors continues to increase, improving the quality of survivorship care may prove to be an effective intervention to ensure proper uptake of screening in this population. We sought to determine which components of survivorship care plans, if any, were associated with guideline adherence to breast and cervical cancer screening among female cancer survivors. Our analysis shows that those survivors who received follow-up instructions were more likely to be guideline adherent to their breast and cervical cancer screening, regardless of whether they received a treatment summary or of the type of physician responsible for their survivorship care. Nevertheless, in both groups about a quarter of cancer survivors had not received these instructions as part of their survivorship care plans; survivors with low income or lack of insurance coverage during cancer care were disproportionately affected.

In order to highlight best practices, a growing body of literature has sought to characterize the health-related behaviors of cancer survivors. There has been conflicting data in the past on screening utilization among cancer survivors, showing both higher [5, 6] and lower [7, 8] screening utilization compared to the general population. However, more recent studies, including two recent meta-analyses showed that overall, cancer survivors are more likely to utilize screening services for breast, cervical, and colorectal cancer [9, 10]. Despite higher screening, cancer survivors still have suboptimal screening rates compared to national guidelines and there are several high-risk groups that receive less screening [11–13]. Our data do show higher adherence to screening guidelines than those reported by the general population [36], and the breast and cervical screening rates found for the cancer survivors in our study are consistent with prior reported rates [11, 18, 37].

Some studies have explored the association between receipt of survivorship care plans and guideline adherence to cancer screening [18–20]. One study investigated the association of written survivorship care plans with health-related behaviors, including the uptake of breast cancer screening among cancer survivors [18]. Receipt of a written SCP plan (both treatment summaries and follow-up instructions) was associated with more routine medical appointments, exercise in the past month, non-smoking status, up-to-date mammograms, but not colorectal cancer screening among survivors [18]. Another study showed that breast and prostate cancer survivors who received a follow-up care plan were more likely to be guideline-adherent with colorectal cancer screening. An additional study demonstrated that survivors receiving both elements of an SCP were more likely to report follow-up checkup and imaging within the last 2 years compared to survivors who received neither element, though cancer screening was not explored [20]. While these studies highlight the benefit of SCPs in promoting screening and other health-related behaviors, neither study explores how individual components of SCPs impact breast and cervical cancer screening, nor do they explore the contribution of social determinants of health. In the context of these prior findings, our study provides evidence that follow-up instructions, a simple, inexpensive component of survivorship care plans, may increase uptake of breast and cervical screening among cancer survivors.

To our knowledge, ours is the first study to assess the potential impact of individual elements of a SCP on breast and cervical cancer screening. While a SCP typically includes both a treatment summary and follow-up instructions, our data demonstrate that these two components are not always given together, with about 75% of participants receiving follow-up instructions and only 50% receiving a written treatment summary. More importantly, a formal, written SCP with both elements is only reported by a minority of survivors, 39% in our dataset, and 25–37% in prior studies [18, 20, 38]. Therefore, previous studies may have missed an important group of survivors who receive follow-up instructions outside of a structured format like a SCP. While the majority of survivors receiving follow-up instructions in our study received written instructions, we found no significant difference in guideline adherence between those receiving written and unwritten instructions. The receipt of follow-up instructions alone, whether written or unwritten or in the setting of a formal SCP or not, may be sufficient to increase cancer screening among cancer survivors.

Our study also found that the type of physician providing survivorship care is not associated with guideline adherence to breast or cervical cancer screening. Primary care providers (PCPs) and oncologists have previously been found to have discordant perceptions of their own roles for cancer survivors’ follow-up, screening, and general preventive health, which could complicate care coordination [39]. Prior studies suggest that both types of providers play important roles for appropriate survivorship care [40, 41], though PCPs may be more prominent in general preventive health [42], and oncologists may contribute more towards cancer surveillance [43]. Our findings suggest that any provider who sees cancer survivors can participate in the promotion of screening behaviors.

Our findings highlight important disparities in the receipt of follow-up instructions among low-income and uninsured cancer survivors. This is particularly important given the disproportionate prevalence, more advanced cancers, and higher cancer mortality rates in these populations [44–46]. Addressing these socioeconomic barriers will be critical in order to increase access to survivorship care. Previous work has shown that having low educational achievement, not having a partner, and being uninsured increases the likelihood of not receiving a SCP [38]. Survivorship care services may not be offered to uninsured patients, especially in light of pre-existing difficulties obtaining reimbursement for survivorship care for insured individuals [38, 47]. Preparing SCPs and coordinating survivorship care is a time-consuming process. However, most U.S insurers only provide reimbursement for a limited number of survivorship care services. For instance, only the time used by physicians (as opposed to non-clinicians) in the preparation of a SCP counts towards reimbursement, although this is equivalent to only a third of the total time used to prepare a SCP [48]. Furthermore, most U.S insurers do not reimburse for certain additional survivorship care services, such as psychosocial support and care planning services [47]. Lobbying for equitable and fair insurance reimbursement for this critical service and creating targeted survivorship programs for uninsured patients is crucial. Patient navigation may be particularly helpful for patients with low health literacy or language barriers [49]. However, other systemic barriers exist, including lack of a trained survivorship care workforce and lack of a cohesive health system to address the multidisciplinary concerns of cancer survivors [47]. Future studies should focus on increasing the dissemination of follow-up instructions to low-income and uninsured populations.

There are several limitations to this study. Since this is a cross-sectional study, findings are limited to a temporal association between elements of survivorship care and cancer screening, and causality cannot be established. Participants self-reported information which may lead to errors. The data are also subject to recall bias because participants are asked about events that occurred in the past, and those who adhered to screening may be more likely to report receiving follow-up care instructions. As the survivorship care plan questions are part of an optional BRFSS module which is only available in a few states every year, the findings may not be generalizable to other states. Additionally, BRFSS does not have more detailed information about treatment summaries and follow-up instructions. There may have been variability in the information for each treatment summary and set of follow-up instructions, and this may have influenced the associations found in this study. Furthermore, this study’s sample is predominantly white, highly educated, partnered, and insured, which may not reflect the experiences of all cancer survivors.

## Conclusion

Receipt of follow-up care instructions, a component of survivorship care plans, is associated with increased adherence to breast and cervical cancer screening guidelines among cancer survivors. However, about a quarter of survivors in the BRFSS did not receive these instructions, and low-income and uninsured survivors were less likely to receive them. The type of doctor providing survivorship care had no association with screening guideline adherence. Future research should focus on identifying avenues to increase dissemination of follow-up instructions, along with the creation of equitable survivorship care programs for expanded access to low-income and uninsured populations.

## Data Availability

The data generated during this study is publicly available at https://www.cdc.gov/brfss/annual_data/annual_data.htm. The specific datasets generated and analyzed in this study are available from the corresponding authors on reasonable request..

## Declarations

### Disclaimers

This research was presented as a poster at the 2023 ASCO Annual Meeting, held in Chicago, IL, June 2 to 6, 2023, and published as an abstract from the meeting.
